# Hypoxia-inducible factor prolyl hydroxylase inhibitors for anemia in heart failure patients: A protocol for systematic review and meta-analysis

**DOI:** 10.1371/journal.pone.0275311

**Published:** 2022-09-28

**Authors:** Hidekatsu Fukuta, Hiromi Hagiwara, Takeshi Kamiya

**Affiliations:** 1 Core Laboratory, Nagoya City University Graduate School of Medical Sciences, Nagoya, Japan; 2 Department of Medical Innovation, Nagoya City University Graduate School of Medical Sciences, Nagoya, Japan; University of Mississippi Medical Center, UNITED STATES

## Abstract

**Background:**

Anemia is common in heart failure (HF) patients with chronic kidney disease (CKD) and is associated with worse outcomes. Iron supplementation improves symptoms and is associated with reduced risk of hospitalization for HF in iron-deficiency HF patients. However, iron deficiency is present in <30% of anemic HF patients. Erythropoiesis stimulating agents (ESAs) improve symptoms but are associated with increased risk of thromboembolic events in anemic HF patients with CKD. Hypoxia-inducible factor prolyl hydroxylase (HIF-PH) inhibitors are a new class of agents for the treatment of anemia. These agents work by stabilizing the HIF complex, thereby stimulating endogenous erythropoietin production. We hypothesized that HIF-PH inhibitors may be associated with reduced risk of cardiovascular outcomes compared with ESAs in anemic HF patients with CKD. Accordingly, we aim to perform the meta-analysis of studies on the efficacy and safety of HIF-PH inhibitors compared with ESAs in anemic HF patients with CKD.

**Methods:**

This meta-analysis will include prospective cohort studies and randomized controlled trials on the effect of HIF-PH inhibitors compared with ESAs in anemic HF patients with CKD. Information of studies will be collected from PubMed, Web of Science, Cochrane Library, and ClinicalTrials.gov. The primary outcome will be cardiovascular death. The secondary outcomes will be all-cause death, hospitalization for HF, HF symptoms, exercise capacity, health-related quality of life, and hemoglobin levels.

**Discussion:**

This meta-analysis will evaluate the effect of HIF-PH inhibitors in anemic HF patients with CKD, providing evidence regarding the use of HIF-PH inhibitors in these patients.

**Systematic review registration:**

INPLASY202230103.

## Introduction

Heart failure (HF) is a major clinical and public health problem. Despite the significant progress in the treatment of HF, the mortality of HF remains high and, in most recent years, ≈50% at 5 years [1].

Chronic kidney disease (CKD) is a common comorbidity in HF patients and HF patients with CKD frequently have anemia [2]. In anemic HF patients, inadequate oxygen supply and impaired oxygen use by skeletal muscle during exercise contribute to poor functional capacity [3]. There is substantial evidence that, as a treatment for anemia, iron supplementation improves symptoms, functional capacity, and quality of life and is associated with reduced risk of hospitalization for HF in iron-deficiency HF patients [4–8]. However, iron deficiency is present in <30% of anemic HF patients and the majority of observed anemia in HF patients results from other factors including inadequate erythropoietin production due to renal insufficiency and intrinsic bone marrow defects [3].

The effect of erythropoiesis stimulating agents (ESAs) in anemic HF patients with CKD has been examined in multiple randomized controlled trials (RCTs) [9–11]. Several meta-analyses of RCTs reported that, compared with placebo, ESA-treatment improved symptoms and had a neutral effect on all-cause mortality and hospitalization for HF but increased the risk of thromboembolic events [12, 13].

Hypoxia-inducible factor (HIF) prolyl hydroxylase (PH) inhibitors are a new class of agents for the treatment of anemia [2, 14]. These agents work by stabilizing the HIF complex, thereby stimulating endogenous erythropoietin production. HIF-PH inhibitors improve iron mobilization to the bone marrow. By inducing considerably lower but more consistent blood erythropoietin levels than ESAs, HIF-PH inhibitors may be associated with fewer adverse cardiovascular effects at comparable hemoglobin levels. Several case reports have reported that HIF-PH inhibitors improved anemia without significant adverse events in HF patients [15, 16]. Although there are several on-going prospective studies on the effect of HIF-PH inhibitors in anemic HF patients with CKD (ClinicalTrials.gov: NCT05053893; UMIN Clinical Trials Registry: 000041651) [17], there is no evidence as to the effect in these patients. We hypothesized that HIF-PH inhibitors may be associated with reduced risk of cardiovascular outcomes compared with ESAs in anemic HF patients with CKD. Accordingly, we aim to perform the meta-analysis of studies on the efficacy and safety of HIF-PH inhibitors compared with ESAs in anemic HF patients with CKD.

## Methods

This study has been registered on International Platform of Registered Systematic Review and Meta-analysis Protocols with registration number of INPLASY202230103 (https://www.doi.org;DOI: 10.37766/inplasy2022.3.0103). This protocol for meta-analysis will be performed according to the Preferred Reporting Items for Systematic Review and Meta-analysis Protocols (PRISMA-P) statement [18].

### Search strategy

The electronic databases for literature search will include PubMed, Web of Science, Cochrane Library, and ClinicalTrials.gov. For search of the eligible studies, the following keywords and Medical Subject Heading will be used: *hypoxia-inducible factor prolyl hydroxylase inhibitor(s)*, r*oxadustat*, *daprodustat*, *vadadustat*, *molidustat*, *desidustat*, *enarodustat*, *and heart failure*. Only articles published in the English language will be included.

### Study design

Prospective cohort studies and RCTs will be included. Retrospective cohort and case–control studies will be excluded.

### Selection criteria

Inclusion criteria for this meta-analysis included: (1) included HF patients with anemia and CKD; (2) prospective cohort studies or RCTs; (3) administration of HIF-PH inhibitors; (4) compared with ESAs; and (5) assessed cardiovascular death, all-cause death, hospitalization for HF, HF symptoms, exercise capacity, health-related quality of life, or hemoglobin levels.

### Outcomes

The primary outcome will be cardiovascular death. The secondary outcomes will be all-cause death, hospitalization for HF, HF symptoms, exercise capacity (6-minute walk distance), health-related quality of life, and hemoglobin levels.

### Data extraction

Information on the study and patient characteristics, methodological quality, intervention strategies, and clinical outcomes will be systematically extracted separately by 2 reviewers. Disagreements will be resolved by consensus. We will contact the corresponding author of eligible studies when insufficient information is available to perform our meta-analysis.

### Quality assessment

The Cochrane Risk of Bias tool will be used to assess quality of RCTs included [19]. The quality of prospective cohort studies will be evaluated by Newcastle-Ottawa Scale tool (http://www.ohri.ca/programs/clinical_epidemiology/oxford.asp). The quality of evidence for the outcomes will be evaluated by use of the Grading of Recommendations Assessment, Development and Evaluation (GRADE) system [20]. The quality of evidence will be evaluated across the domains of risk of bias, consistency, directness, precision, and publication bias.

### Meta-analysis sample size calculation

We will determine the required meta-analysis sample size as previously reported [21]. Briefly, after 5 studies report the results of the primary endpoint (cardiovascular death), we will determine the required meta-analysis information size for detecting a reported relative risk reduction in HIF-PH inhibitors group (the median relative risk reduction across trials). We will calculate the information size required to yield meta-analytic evidence based on an alpha = 5% significance level, and beta = 20% (80% power). To evaluate if the observed effects in the two treatments groups differ significantly, we will use a standardized test statistic (Z-statistic) which can be transformed to a P-value. Z-statistics that lie outside the interval −1.96 to 1.96 correspond to P-values smaller than 0.05. The monitoring boundaries will be applied every time one or more studies are added up until the point where the number of patients in the meta-analysis surpasses the required meta-analysis information size.

If the required meta-analysis sample size is not available, a systematic narrative synthesis will be provided with information presented in the text and tables to summarize and explain the characteristics and findings of the included studies. The narrative synthesis will explore the relationship and findings both within and between the included studies, in compliance with the guidance from the Centre for Reviews and Dissemination [18].

### Statistical analysis

For morbidity and mortality, hazard ratios will be pooled. For continuous outcomes, the effect size for the intervention will be calculated by the difference between the means of the intervention and control groups at the end of the intervention. If the outcome is measured on the same scale, the weighted mean difference and 95% confidence interval (CI) will be calculated. Otherwise, the standardized mean difference and 95% CI will be calculated. For each outcome, heterogeneity will be assessed using the Cochran’s Q and *I*^2^ statistic; for the Cochran’s Q and *I*^2^ statistic, a p value of <0.1 and *I*^2^>50%, will be considered significant, respectively. When there is significant heterogeneity, the data will be pooled using a random-effects model, otherwise a fixed-effects model will be used. Publication bias will be assessed graphically using a funnel plot and mathematically using Egger test. For these analyses, Comprehensive Meta Analysis Software version 2 (Biostat, Englewood, NJ, USA) and STATA 16 software (Stata Corp LP, TX, USA) will be used.

### Sensitivity analysis

Subgroup analysis stratified by study design (RCT or prospective cohort study) will be performed. Meta-regression will be used to determine whether the effect of HIF-PH inhibitors will be confounded by baseline clinical characteristics such as age, sex, New York Heart Association functional class, CKD stage, and hemoglobin levels.

### Ethical issues

This meta-analysis is a literature study. Ethical approval is not required because this meta-analysis will not involve any subject directly.

## Discussion

In the recent guidelines, HIF-PH inhibitors are recommended as alternatives to ESAs in correcting and maintaining hemoglobin level for renal anemia in CKD patients [22]. However, there is no evidence as to the efficacy and safety of HIF-PH inhibitors compare with ESAs in anemic HF patients with CKD.

Several experimental studies have reported that HIF-PH inhibitors have advantages over ESAs. Specifically, it is suggested that exogenous erythropoietin generated by ESAs can largely increase C-terminal fibroblast growth factor 23 (FGF23) levels [23], which is significantly associated with left ventricular hypertrophy and an increased risk of mortality. In contrast, HIF-PH inhibitors have been reported to decrease FGF23 levels in an animal model of CKD [24]. Furthermore, HIF-PH inhibitors have been reported to reduce myocardial ischemia reperfusion injury in mice [25].

To the best of our knowledge, this is the first meta-analysis protocol on the effect of HIF-PH inhibitors in anemic HF patients with CKD. The results will evaluate whether HIF-PH inhibitors are beneficial for anemic HF patients with CKD, providing evidence regarding the use of HIF-PH inhibitors in these patients.

## Abbreviations

ESAs: erythropoiesis stimulating agents
GRADE: Grading of Recommendations Assessment, Development and Evaluation
HF: heart failure
HIF-PH: hypoxia-inducible factor prolyl hydroxylase
PRISMA-P: Preferred Reporting Items for Systematic Review and Meta-analysis Protocols
RCTs: randomized controlled trials
